# Ultrasound‐Assisted NADES‐Based Extraction of Spirulina Bioactives: Bioaccessibility and Intestinal Permeability Assessment Using an In Vitro Digestion/Caco‐2 Cell Model and Food Application

**DOI:** 10.1111/1750-3841.71289

**Published:** 2026-07-17

**Authors:** Gamze Toydemir, Ilayda Sanli, Gulay Ozkan, Esra Capanoglu

**Affiliations:** ^1^ Department of Food Engineering Faculty of Engineering Alanya Alaaddin Keykubat University Alanya Antalya Türkiye; ^2^ Department of Food Engineering, Faculty of Chemical and Metallurgical Engineering Istanbul Technical University Maslak Istanbul Türkiye

**Keywords:** antioxidant, Caco‐2 cell model, in vitro bioaccessibility, kefir, NADES, Spirulina

## Abstract

In this study, ultrasound‐assisted extraction was applied using eight NADES formulations to recover Spirulina antioxidants, and the bioaccessibility and apparent basolateral recovery of phenolics in the most potent extracts were evaluated, both in the original extracts and after their incorporation into a kefir matrix, through an in vitro digestion/Caco‐2 cell model. The extracts obtained with glucose:glycerol exhibited the highest pigment and total phenolic content (TPC), while betaine:glycerol and choline chloride:citric acid extracts exhibited the highest antioxidant capacity. Although Spirulina‐fortified kefir samples had significantly lower (> 90% reduction) TPCs than individual Spirulina samples, they exerted increased apparent in vitro bioaccessibility of phenolics during digestion, with the highest value reaching 355% for kefir incorporated with the glucose:glycerol extract. This elevated response is likely associated with matrix‐related release, dissociation, and transformation of Folin‐reactive compounds during digestion rather than reflecting actual intestinal absorption of phenolic compounds. Relatively higher apparent basolateral recovery of antioxidants in the Caco‐2 cell model was also achieved in some Spirulina‐fortified kefir samples. These findings highlight the potential use of NADES with ultrasonication for the enhanced recovery of Spirulina antioxidants and also revealed that kefir can serve as an effective carrier matrix for improved gastrointestinal utilization of Spirulina‐derived bioactives.

**Practical Applications:**

This study shows that kefir can be considered a promising carrier matrix for Spirulina antioxidants. Since Spirulina is widely used as a functional food ingredient, its incorporation into kefir may offer a commercially attractive strategy for producing probiotic beverages with added antioxidant and natural pigment value. The present findings may assist the food industry in designing more sustainable and value‐added products using microalgae‐based ingredients and alternative extraction systems.

## 1 Introduction

Among microalgae, Spirulina (*Limnospira platensis)* has attracted considerable attention as a sustainable source of various macronutrients, pigments (like phycocyanin, chlorophyll, and carotenoid), and polyphenols and is referred to as a “superfood” due to this distinctive profile (Haoujar et al. 2022; Hilali et al. 2024 ; Raczyk et al. 2022). Spirulina exhibits various biological functions, such as antioxidant, antimicrobial, and anti‐inflammatory activities, and has the potential to be used as a malnutrition suppressant (AlFadhly et al. 2022; Spínola et al. 2024). Although this unique bioactivity makes Spirulina a potent raw ingredient to develop functional foods (Seyidoglu et al. 2017; Yang et al. 2019), its unpleasant fishy/earthy odor, deep bluish‐green color, and astringent taste, which are difficult to mask in food formulations, significantly limit its use and wide‐scale acceptance in food products (Marjanović, et al. 2024; Shahid et al. 2025). At this point, isolating Spirulina bioactives for further use in foods may be a promising approach to overcome these sensorial challenges and to enhance their health‐related benefits (Marjanović, et al. 2024).

The recovery of bioactive compounds from microalgae highly depends on the applied extraction method, in addition to the solvent used for extraction. Various conventional extraction methods, such as maceration, hydrodistillation, steam distillation, and Soxhlet extraction, along with conventional solvents like n‐hexane, ethyl acetate, methanol, and acetone, have been employed to extract bioactive compounds (Garcia‐Vaquero et al. 2020; Picot‐Allain et al. 2021). However, conventional extraction has been well‐addressed to have several adverse effects, including being time‐consuming, having low extraction efficiency, causing decomposition of thermolabile compounds, and the use of toxic organic solvents (Garcia‐Vaquero et al. 2020; Hilali et al. 2024; Martí‐Quijal et al. 2021; Picot‐Allain et al. 2021). In this regard, green extraction methods like ultrasound‐assisted extraction (UAE) have emerged as a promising alternative method due to their environmental friendliness, higher extraction efficiency, and lower solvent usage (Benvenutti et al. 2019; Martins et al. 2023; Rodríguez‐Juan et al. 2021).

Besides green technologies, green extraction solvents, known as natural deep eutectic solvents (NADESs), have recently gained increasing interest as promising alternatives to conventional solvents. NADESs are formed by mixing hydrogen bond acceptors (HBAs), such as quaternary ammonium salts (i.e., choline chloride and betaine) and hydrogen bond donors (HBDs), such as carbohydrates, amines, organic acids, or alcohols, which are naturally present in all cells and organisms. The resulting eutectic mixture has a lower melting point than its individual components, giving rise to a liquid system with a broad liquid‐phase range (Fanali et al. 2020; Ozkan 2024). NADESs offer several advantages, such as high biodegradability, food‐grade safety, and improved extraction efficiency and bioavailability of the final extracts (Hilali et al. 2022). Additionally, NADES‐extracted bioactives exhibit enhanced stability during and after the extraction process, making NADESs promising for application in novel functional foods (González‐Laredo et al. 2023). These properties have led to increasing interest in food‐grade NADESs for their direct incorporation into food matrices, where they can act not only as extractants but also as stabilizers and preservatives (Martins et al. 2025).

The in vitro antioxidant activity measured in non‐digested foods may overestimate the actual in vivo antioxidant potential of complex food matrices (Martinelli et al. 2021). Thus, in order to evaluate the health‐related effects and functional food potential of Spirulina NADES extracts, one of the primary analyses is to determine the changes in Spirulina antioxidants during digestion. Despite the growing interest in NADES for recovering bioactive compounds from various natural sources, studies on Spirulina remain limited, particularly regarding the post‐digestion behavior and transepithelial transport of its bioactive compounds (Li et al. 2023). Simulated in vitro gastrointestinal digestion followed by the transport studies using the human intestinal Caco‐2 cell system is a well‐established, cost‐effective approach for mimicking physiological conditions and predicting the intestinal transport of bioactive food compounds (Wu et al. 2017). In addition, in vitro antioxidant assays have previously been used to determine the antioxidant activities of food samples before and after simulated gastrointestinal digestion (Chandrasekara and Shahidi 2012; Erdem et al. 2025; Prado Massarioli et al. 2023) and after Caco‐2 cell transport (Duan et al. 2025; Prado Massarioli et al. 2023). Moreover, Liao et al. (2020) reported strong correlations between cellular antioxidant activity measurements performed on Georgia peach extracts using a Caco‐2 cell model and spectrophotometric TPC and ORAC values obtained for those samples.

**FIGURE 1:**
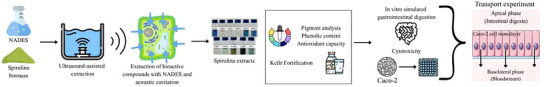
Schematic representation of the experimental workflow including ultrasound‐assisted extraction of Spirulina, incorporation into kefir, in vitro gastrointestinal digestion, and Caco‐2 cell transport analysis.

Although recent studies have demonstrated the potential of NADES for the recovery of Spirulina bioactives, these studies have mainly focused on extraction efficiency and compositional characterization (Della Posta et al. 2025; Lin et al. 2025). The novelty of the present study lies in combining NADES‐based UAE of Spirulina antioxidants with their incorporation into a kefir matrix and evaluating their fate during simulated gastrointestinal digestion and differentiated Caco‐2 cell transport. Accordingly, this study not only compared the extraction performance of NADES with conventional solvents (ethanol, methanol, and water) in terms of total phenolic content (TPC), pigment content, and antioxidant activity, and further investigated the apparent bioaccessibility and intestinal permeability of Spirulina antioxidants both as original NADES extracts and after their incorporation into a kefir matrix, as summarized in Figure 1.

## 2 Materials and Methods

### 2.1 Chemicals and Spirulina Biomass

Spirulina powder was obtained from a local commercial supplier (Sepe Natural Organic Products Industry and Trade Inc., İzmir, Türkiye) and stored at room temperature and in dry conditions before analysis. The components used to prepare NADESs, including choline chloride, glucose, glycerol, betaine, lactic acid, acetic acid, and citric acid, were supplied from Sigma‐Aldrich (Steinheim, Germany). The Caco‐2 cell line was obtained from Yeditepe University Department of Medical Genetics Research Group (Istanbul, Türkiye). All chemicals for cell culture experiments were obtained from Capricorn Scientific (Ebsdorfergrund, Germany). All other reagents were of analytical grade and purchased from Sigma‐Aldrich (Steinheim, Germany).

### 2.2 NADES Preparation and Analysis

In the preparation of NADES, choline chloride and betaine were used as the HBAs. Lactic acid, citric acid, and acetic acid were used as HBDs, while glucose and glycerol were used as both HBAs and HBDs. The NADESs were prepared by mixing the represented ratios of HBAs and HBDs, and water in Table S1. As the NADES system is well‐studied to have an extensive hydrogen bonding network (Dai et al. 2015; Hikmawanti et al. 2021), water was included in all NADES solutions in order to reduce the viscosity of the final solvent mixture for better extraction of the intended compounds with improved mass transfer. All the mixtures were heated at 80°C with subsequent stirring until homogenous and colorless liquids were obtained.

The density measurements of the NADES solutions were performed by weighing 1 mL of NADES filled in flasks on an analytical balance at room temperature. The following equation was applied for density calculation: *ρ* = *m*_NADES_/*V*_NADES_.

The physicochemical properties (pH and viscosity) of NADESs 2, 3, 5, 7, and 8 were previously characterized under identical preparation conditions (Karabulut et al. 2025), and NADES 4 has been comprehensively characterized in a previous study by Rocha et al. (2023). Furthermore, recent literature provides additional physicochemical characterization of both sugar‐based and organic acid‐based NADES systems (Jiménez‐Ortega et al. 2025; Lomba et al. 2023; Tarikuzzaman et al. 2024).

### 2.3 Ultrasound‐Assisted Extraction of Bioactives From Spirulina

UAE was applied to Spirulina biomass either by using conventional solvents, including methanol:water (80:20, v/v), ethanol:water (80:20, v/v), and water, or NADES solutions that were prepared as specified in Table S1. Extraction was performed by using the procedure described previously (Ozkan 2024). Sonication was performed in an ultrasonic bath (VWR USC900TH, Radnor, PA, USA) operating at 45 kHz and 100% power (600 W) for 15 min. For a treated sample volume of 100 mL, the nominal power density corresponded to 6000 W/L and the energy density to 5400 J/mL. The bath temperature was maintained below 30°C throughout sonication to minimize thermal degradation. Supernatants were collected and stored at 4°C until further analysis.

### 2.4 Identification and Quantification of Phenolic Compounds by HPLC

Phenolic compounds were identified and quantified according to the method described previously by Capanoglu et al. (2008). Waters 2695 HPLC system equipped with a PDA detector (Waters 2996) was used. Separation was carried out on a Supelcosil LC‐18 column (25 cm × 4.60 mm, 5 µm; Sigma‐Aldrich, Steinheim, Germany) using TFA/Milli‐Q water (0.1%, v/v; solvent A) and TFA/acetonitrile (0.1%, v/v; solvent B) as the mobile phases, at a flow rate of 1 mL/min and an injection volume of 10 µL. Detection was performed at 280 nm for hydroxybenzoic acids, 312 nm for hydroxycinnamic acids, and 360 nm for flavonoids. Phenolic compounds were quantified using external calibration curves prepared from authentic standards in the range of 1.55‐200 mg/L, with all curves showing good linearity (*R* ^2^ ≥ 0.999). The gradient program was as follows: 5% B at 0 min, 35% B at 45 min, 75% B at 47 min, and re‐equilibration to initial conditions at 54 min. The results were expressed as mg/100 mL extract.

### 2.5 Determination of Bioactive Properties

#### 2.5.1 Pigment Analysis

The concentrations of chlorophylls a and b, carotenoids, and phycocyanin were determined in all Spirulina extracts spectrophotometrically (UV‐visible spectrophotometer; Synergy HT; BioTek Instruments, Winooski, VT, USA). The extracts were monitored for the highest absorbance at a specific wavelength spectrum, and the concentrations of the bioactive pigments were calculated by using the following equations (Martins et al. 2023):

$$C_{chlorophylla+b}=17.76A_{646.6}-7.34A_{663.6}$$

$$C_{carotenoids}=4.69A_{440}-0.267C_{chlorophylla+b}$$

$$C_{phycocyanin}=\left(A_{620}-0.474A_{652}\right)/5.34$$

in which C_chlorophyll a+b_, C_carotenoids_, and C_phycocyanin_ are the concentrations of chlorophyll a and b, total carotenoids, and phycocyanin in mg/100 mL of extract. Additionally, *A*_646.6_, *A*_663.6_, *A*_440_, *A*_620_, and *A*_652_ represent the absorbance values at 646.6, 663.6, 440, 620, and 652 nm, respectively. The blank absorbance values for each solvent are presented in Table S2.

#### 2.5.2 Analysis of Total Phenolic Content

The TPCs of the Spirulina extracts obtained by conventional solvents or NADES solutions were determined using the Folin–Ciocalteu method as described previously (Singleton and Rossi 1965). Briefly, 20 µL of extract, 1.58 mL of distilled water, 100 µL of Folin's reagent, and 300 µL of sodium carbonate (20%, w/v) were mixed, and subsequently, the mixture was vortexed and heated at 40°C for 90 min. Absorbance values were measured at 725 nm using a UV‐visible spectrophotometer (BioTek Instruments, Winooski, USA). Pure NADES solutions or conventional solvents were used as blanks for the respective Spirulina extracts. The results were expressed as mg GAE (gallic acid equivalents)/100 mL extract by using a gallic acid standard curve (*R* ^2^ = 0.995; linear range: 0.01–0.4 mg/mL). All measurements were conducted in triplicate, and the measured absorbance values of the corresponding solvent blanks that were pure NADES and conventional solvents are given in Table S2.

#### 2.5.3 Determination of Total Antioxidant Capacity by DPPH and CUPRAC Assays

The total antioxidant capacity (TAC) of the Spirulina extracts obtained by conventional solvents or NADES solutions was determined using DPPH (2,2‐diphenyl‐1‐picrylhydrazyl) (Kumaran and Joel Karunakaran 2006) and CUPRAC (cupric ion reducing antioxidant capacity) (Apak et al. 2004) assays.

In DPPH assay, 10 µL of extract was mixed with 200 µL of 0.1 mM DPPH solution (in 75% (v/v) methanol) and incubated in the dark for 30 min. Following incubation, the absorbance was recorded at 517 nm using a spectrophotometer.

For the CUPRAC assay, copper (II) chloride solution (0.01 mM) was prepared in distilled water, while the neocuproine solution (7.5 mM) was prepared in 96% ethanol. Then, 7 µL of the extract was added to 280 µL of the reaction mixture containing 70 µL of copper (II) chloride solution, 70 µL of neocuproine solution, 70 µL of ammonium acetate buffer solution (pH 7.0), and 70 µL of distilled water. The mixture was incubated in the dark for 30 min, and the absorbance was measured at 450 nm using a spectrophotometer.

Pure NADES solutions or conventional solvents were used as blanks for the respective Spirulina extracts. The results were expressed as mg Trolox equivalents (TE)/100 mL extract based on the calculations performed using a Trolox calibration curve (*R* ^2^ ≥ 0.996; linear range: 0.01–0.4 mg/mL). All measurements were conducted in triplicate, and the measured absorbance values of the corresponding pure NADES and conventional solvent blanks are given in Table S2.

### 2.6 Food Application

Among the Spirulina extracts prepared as described in Section 2.3, only four of them, including NADES 1, NADES 5, NADES 8, and water extracts, were selected for application in kefir based on the bioactive analysis results (Section 2.5). Each of the selected Spirulina extracts was directly incorporated into the kefir matrix (5%, v/v) to obtain a fortified kefir formulation. The commercial plain kefir (Altınkılıç Food and Milk Industry and Trade Inc., Türkiye) contained 3.7 g protein, 2.9 g fat, and 3.7 g carbohydrates per 100 mL. The product exhibited a typical pH range of 4.2–4.6. For the extraction of fortified kefir samples, 2.5 mL of each sample was diluted with 70% methanol (v/v) to a final volume of 20 mL. The mixtures were incubated on a magnetic stirrer at 20°C for 4 h in the dark to prevent photodegradation, followed by centrifugation at 3000 rpm for 15 min at 4°C. The supernatants were collected and stored at 4°C for subsequent analyses.

### 2.7 In Vitro Simulated Gastrointestinal Digestion

The Spirulina extracts selected for food application (NADES 1, NADES 5, NADES 8, and water extracts) and kefir samples fortified with these selected Spirulina extracts (Kefir + NADES 1, Kefir + NADES 5, Kefir + NADES 8, and Kefir + water) were subjected to a simulated gastrointestinal digestion model, which was performed according to the protocol described by Minekus et al. (2014). The salivary, gastric, and intestinal electrolyte solutions were prepared as outlined in the protocol. Blank samples, which were the respective NADES solutions without the Spirulina biomass, were subjected to identical digestion procedures and were used to correct for potential interferences that may arise from NADES components and digestive fluids. All samples collected from the simulated in vitro gastric and intestinal digestion phases were centrifuged at 23,000 *g* for 30 min at 4°C, and the resulting supernatants were stored at −18°C until further spectrophotometric measurements of TPC and TAC, as detailed in Sections 2.5.2 and 2.5.3. For calculating the bioaccessibility index, the following equation was used: Bioaccessibility index (%) = (TPC or TAC values after digestion/TPC or TAC values before digestion) × 100.

### 2.8 Cytotoxicity and Transport Experiments With Caco‐2 Cell Culture Model

Caco‐2 cells, as an intestinal epithelial model, were cultivated in a growth medium consisting of 78% Dulbecco's modified Eagle's medium, 20% fetal bovine serum, 1% nonessential amino acids, and 1% penicillin/streptomycin (Ozkan et al. 2022). The cells were maintained at 37°C with 95% humidity and 5% CO_2_. Cells were subcultured every 2 days and used at passage 40. The experiments were conducted in accordance with institutional biosafety regulations. As no human subjects or primary tissues were involved, ethical committee approval is not required.

For the cytotoxicity test, the cells were seeded into 96‐well plates (0.32 cm^2^/well, Sarstedt, Nümbrecht, Germany) (200 µL/well and 2500 cells/well [approximately 7.8 × 10^3^ cells/cm^2^]) (De Vreese et al. 2016) and cultured for 14 days with medium replacement every 2–3 days. Note that 200 µL of intestinal phase samples was diluted (1/2, 1/5, 1/10, and 1/20 [v/v]) in Hanks′ Balanced Salt solution (HBSS), and a control group (no sample) was also included. Following a 4‐h waiting period, 20 µL of 3‐(4,5‐dimethylthiazol‐2‐yl)‐2,5‐diphenyltetrazolium bromide (MTT) solution (5 mg MTT/mL PBS) was applied after 100 µL of culture media was removed. After 2 h of incubation, the media was taken out, and the 96‐well plate was dried. The formazan crystals were dissolved by adding 200 µL of dimethyl sulfoxide (DMSO), and the absorbance was determined at 570 nm using a spectrophotometer (BioTek Instruments, Winooski, USA).

For the transport experiment, Caco‐2 cells were seeded onto 12‐transwell inserts (0.4 µm pore diameter, 24 mm insert, Sarstedt, Nümbrecht, Germany). The apical side received 0.5 mL of growth medium with a density of 1 × 10^5^ cells/well (approximately 2.2 × 10^4^ cells/cm^2^), whereas the basolateral side received 1 mL of growth medium. The cells underwent differentiation for 21 days, and the growth medium was changed every 2 days. The transepithelial electrical resistance (TEER value) was measured to assess the quality of Caco‐2 cells during this differentiation using a Millicell ERS‐2 Volt‐Ohm Meter (Millipore, Bedford, MA, USA). Then, only differentiated cells were employed for further steps. In accordance with Ozkan et al. (2022), 500 µL of HBSS was added to the apical side and 1 mL to the basal side to maintain cell health, and the medium was incubated for 1 h. The appropriate dilutions that were determined in the MTT assay were used for further steps. Each of the diluted samples was added to the apical side as 500 µL, and 1 mL of HBSS was applied to the basal side; then, 12‐transwell plates were incubated under the same growth conditions for 4 h. After the incubation, samples were collected from the basolateral compartments to evaluate spectrophotometric assays (TPC, DPPH, and CUPRAC). The ratio between the intestinal phase of each sample and the corresponding basolateral fraction was used to calculate the apparent basolateral recovery (%) of phenolic‐related compounds and antioxidant response.

TEER values were measured before and after the incubation period to verify monolayer integrity. Before incubation, TEER values were between 230 and 283 Ω·cm^2^. After incubation, HBSS was replaced with the growth medium and incubated for 20 h, then TEER values were recorded between 213 and 271 Ω·cm^2^. TEER measurements were corrected using the blank (medium), which was 11 ± 2 Ω·cm^2^. The observed values were acceptable and indicated no significant irreversible damage to the cell monolayer (Table S3).

### 2.9 Statistical Analysis

Extraction was performed in two independent batches as biological replicates. For each batch, spectrophotometric measurements were conducted in triplicate as analytical replicates. Caco‐2 viability and transport experiments were performed in triplicate wells (biological replicates). The results are expressed as mean values ± standard deviations of all analytical replicates. Statistical analysis was carried out using SPSS software (version 20.0, SPSS Inc., Chicago, IL, USA). Differences between treatments were evaluated and compared to each other by applying one‐way analysis of variance (ANOVA) followed by Tukey's post hoc test (*p* < 0.05). For digestion experiments, two‐way ANOVA was performed separately for each extract to evaluate the main effects of matrix (extract vs. kefir), digestion stage (undigested, gastric, intestinal), and their interaction. Significant matrix × stage interactions (*p* < 0.001) indicated that the kefir matrix modulated phenolic and antioxidant behavior during simulated digestion. Exact *p*‐values are presented in Table S4.

## 3 Results and Discussion

### 3.1 Density of NADES

The density of NADES is a supportive physicochemical characterization data, since it may primarily influence feasibility and extraction behavior (Karabulut et al. 2025). According to the results obtained from density measurements of NADES, choline chloride:acetic acid:water (1:1:2) (NADES 7) had the minimum value of 1041 kg/m^3^, while betaine:glycerol:glucose:water (1:2:4:0.5) (NADES 6) had the maximum value of 1190 kg/m^3^. The detailed density data are provided in Table S1. These values are consistent with the values reported in the previous literature to range in between 1.0 and 1.35 g/cm^3^ at room temperature (Saini et al. 2022).

### 3.2 Recovery of Spirulina Bioactives

The amounts of pigments, TPC, and TAC of the Spirulina extracts (Figure S1) obtained via the use of eight different NADESs were determined and compared to the values obtained via the use of conventional solvents (Table 1). In addition, chromatographic characterization indicated that the extracts contained several major phenolic compounds, including gallic acid, syringic acid, (−)‐epicatechin, rutin, quercetin, apigenin, chlorogenic acid, and ferulic acid, varying based on the extraction solvent used (Table S5).

**TABLE 1:** The contents of pigments, total phenolics, and total antioxidant capacity of the Spirulina extracts.

Solvent	Chlorophyll (mg/100 mL)	Carotenoid (mg/100 mL)	Phycocyanin (mg/100 mL)	TPC (mg GAE/100 mL)	CUPRAC (mg TE/100 mL)	DPPH (mg TE/100 mL)
NADES 1	27.67 ± 1.34^a^	ND	519.98 ± 22.15^a^	404.29 ± 48.49^a^	327.20 ± 36.54^ab^	85.87 ± 10.05^a^
NADES 2	1.63 ± 0.09^de^	1.44 ± 0.15^d^	16.30 ± 1.21^e^	142.89 ± 15.97^c^	102.21 ± 13.13^d^	36.15 ± 6.4^cd^
NADES 3	1.88 ± 0.03^de^	1.26 ± 0.03^d^	19.28 ± 0.38^e^	29.17 ± 8.72^e^	89.13 ± 1.94^d^	21.42 ± 4.44^de^
NADES 4	1.35 ± 0.03^e^	0.91 ± 0.02^d^	13.83 ± 0.16^e^	67.38 ± 3.02^de^	86.35 ± 2.74^d^	10.53 ± 1.00^e^
NADES 5	22.11 ± 1.89^b^	ND	337.59 ± 9.13^b^	334.76 ± 14.30^a^	355.64 ± 31.55^a^	43.88 ± 7.57^c^
NADES 6	16.45 ± 1.46^b^	ND	504.24 ± 25.34^a^	143.74 ± 12.69^c^	261.43 ± 22.27^c^	64.16 ± 4.34^b^
NADES 7	1.28 ± 0.07^e^	1.18 ± 0.18^d^	14.22 ± 0.85^e^	134.26 ± 14.93^cd^	119.43 ± 7.51^d^	22.79 ± 7.20^de^
NADES 8	4.90 ± 2.18^d^	1.80 ± 0.29^cd^	36.30 ± 16.13^e^	303.64 ± 6.97^b^	88.03 ± 3.47^d^	102.56 ± 15.99^a^
Methanol	ND	16.25 ± 1.78^b^	17.85 ± 2.84^e^	61.39 ± 4.61^e^	271.74 ± 37.09^c^	46.05 ± 3.50^bc^
Ethanol	28.07 ± 4.75^a^	24.26 ± 1.11^a^	124.42 ± 4.92^d^	88.77 ± 6.71^cde^	337.08 ± 39.88^ab^	37.37 ± 2.64^cd^
Water	10.61 ± 2.11^c^	3.54 ± 0.96^c^	142.96 ± 23.11^c^	319.98 ± 9.06^b^	288.07 ± 27.92^bc^	33.48 ± 3.35^cd^

*Note*: Different lowercase letters within the same column indicate statistically significant differences among samples (*p* < 0.05).

The chlorophyll content of Spirulina extracts differed considerably depending on the solvent used and had the minimum value for choline chloride:acetic acid (1:1) (NADES 7) (1.28 mg/100 mL) and the maximum value for ethanol (28.07 mg/100 mL), while no chlorophyll was detected in the methanolic extract. The extract prepared with glucose:glycerol (1:2) (NADES 1) had comparable, statistically not significantly different, chlorophyll content with ethanolic extract (*p* > 0.05). Betaine:glycerol (1:2) (NADES 5) and betaine:glycerol:glucose (1:2:4) (NADES 6) extracts had chlorophyll contents that were 79% and 59% of the ethanolic extract, respectively (*p* < 0.05). These results indicate the use of NADES, particularly those prepared with glycerol, as an effective alternative to the conventional solvents for the extraction of chlorophyll pigment. In another related study, glycerol‐ and glucose‐based NADESs were also reported to give better yields in chlorophyll extraction, providing the highest values in the glucose:glycerol NADES prepared in the molar ratio of 1:2. These findings suggest that certain NADES systems facilitate interaction with the O‐H and C = O groups present in the structure of chlorophyll and highlight the importance of the molar ratios of the individual HBA and HBD used in the preparation of NADES (Rumiyati et al. 2024).

The ethanolic extract also had the highest carotenoid content (24.26 mg/100 mL), followed by the methanolic (16.25 mg/100 mL) and water (3.54 mg/100 mL) extracts, all of which were significantly higher than the NADES extracts. Among the NADES systems, the highest carotenoid content was measured with choline chloride:citric acid (1:2) (NADES 8) (1.80 mg/100 mL), with a value of only 7% of the ethanolic extract, while carotenoids were not detected or present at trace levels in most glucose‐ and/or glycerol‐based NADES extracts. These results clearly demonstrated that the hydrophilic NADES was far less efficient than the conventional solvents in carotenoid extraction, likely due to the nonpolar and lipophilic nature of carotenoids. The relatively higher performance of choline chloride:citric acid (NADES 8) may be related to the lower viscosity and acidic nature of this solvent system, which may facilitate mass transfer and partial cell disruption (Jauregi et al. 2024). On the other hand, sugar‐based NADES, which are suitable for the recovery of polar components, have been reported to exert low or no efficiency in carotenoid extraction (Koh et al. 2023). Thus, hydrophobic NADES systems, such as terpene (menthol and thymol)‐ or fatty acid (decanoic and dodecanoic acid)‐based formulations, may offer more suitable alternatives for the recovery of carotenoids and other lipid‐soluble bioactives from Spirulina in future studies (Cannavacciuolo et al. 2022; Devi et al. 2023).

For phycocyanin, the highest results were obtained with glucose:glycerol (1:2) (NADES 1) (519.98 mg/100 mL) and betaine:glycerol:glucose (1:2:4) (NADES 6) (504.24 mg/100 mL) extracts, which were not statistically significantly different from each other (*p* > 0.05). These observations indicated that a more efficient phycocyanin extraction was achieved with NADES systems, particularly sugar‐based ones, compared to conventional solvents, which can be attributed to the sugar–protein interactions that could promote the thermal stability of this pigment through polymerization, as highlighted by the study of Lin et al. (2025) and Hilali et al. (2022). Additionally, organic acid‐based NADES (like NADES 7 and NADES 8) had lower pH values that could negatively influence the stability of phycocyanin pigment. As phycocyanin is stable near neutral pH and prone to structural degradation under acidic conditions, such solvents may explain the reduced extraction efficiency observed in organic acid‐based systems (Lin et al. 2025; Mrotek et al. 2024).

Depending on the solvent type, the TPC of the Spirulina extracts ranged between 29.17 and 404.29 mg GAE/100 mL. In general, NADESs were found to be superior for phenolic extraction compared to conventional solvents. The highest TPC values were measured for glucose:glycerol (1:2) (NADES 1) (404.29 mg GAE/100 mL) and betaine:glycerol (1:2) (NADES 5) (334.76 mg GAE/100 mL) extracts. The water extract had significantly lower (*p* < 0.05) TPC than glucose:glycerol (1:2) (NADES 1) extract corresponding to 79% of the TPC of the glucose:glycerol (1:2) (NADES 1) extract. The high number of hydroxyl and carboxyl groups in sugar and polyol‐based NADESs may improve the solubility of phenolic compounds via enhanced hydrogen bonding. In addition, the greater polarity of these solvents makes them more effective for the recovery of polar phenolics (Ceylan and Sezen 2024; Hikmawanti et al. 2021). In parallel with the present study, Boyle and Verghese (2024) also reported phenolic contents that were 3.5‐fold higher in water extracts of Spirulina compared to the values obtained in the 80% EtOH extracts. In another related study, Zannou et al. (2022) compared NADES systems with conventional solvents in their efficiency to extract phenolic compounds from borage flowers and reported that choline chloride/glycerol (1:2) with 20% water (27.76 mg GAE/g) provided a higher yield compared to ethanol (10.08 mg GAE/g) and methanol (20.33 mg GAE/g). Similarly, Cokdinleyen et al. (2024) also observed a higher phenolic content (approximately 15 mg GAE/g) in *Palmaria palmata* extracts that were obtained using choline chloride:glycerol (1:2, 60% water) NADES system than the extracts obtained via the use of 70% ethanol (approximately 10 mg GAE/g). These findings may be attributed to the increased hydrogen bond interactions with NADES and the target compounds, resulting in an enhanced extraction yield. Furthermore, betaine‐based NADES has been reported to be superior for phenolic extraction with the properties of mild alkalinity and enhanced hydrogen bonding capacity upon water addition, leading to the partial ionization of phenolic compounds and their enhanced mass transfer (Tamayo‐Rincón et al. 2025).

Antioxidant properties of the extracts were evaluated using CUPRAC and DPPH assays. In the CUPRAC assay, the highest antioxidant capacity was obtained for betaine:glycerol (1:2) (NADES 5) extract (355.64 mg TE/100 mL). The ethanol (337.08 mg TE/100 mL) and glucose:glycerol (1:2) (NADES 1) (327.20 mg TE/100 mL) extracts showed comparable antioxidant capacities and were not significantly different (*p* > 0.05). In the DPPH assay, choline chloride:citric acid (1:2) (NADES 8) extract exhibited the highest radical scavenging activity (102.56 mg TE/100 mL), which was followed by glucose:glycerol (1:2) (NADES 1) extract (85.87 mg TE/100 mL), with no significant difference between the two extracts (*p* > 0.05). In both CUPRAC and DPPH assays, glycerol:lactic acid (1:1) (NADES 4) extract had the lowest antioxidant response. Notably, glycerol:lactic acid (1:1) (NADES 4) extract had significantly lower amounts of pigments and TPC. In a recent study, NADES extracts of *P. palmata* were also demonstrated to exert higher antioxidant activity than ethanol extracts, highlighting the superior performance of NADES systems (Cokdinleyen et al. 2024). Likewise, Zannou et al. (2022) reported that the use of a choline chloride:glycerol (1:2) + 20% water NADES system in borage flower extraction yielded a higher DPPH value of 146.92 mmol TE/g, compared with conventional solvents (methanol, ethanol, and water) (48.35–24.61 mmol TE/g). In the same study, FRAP values were also higher (939.85 mmol TE/g) in the NADES extracts than in the conventional solvent extracts (444.73–665.53 mmol TE/g), confirming that NADES systems outperform conventional solvents across different antioxidant capacity assays.

### 3.3 Evaluation of Phenolic Stability and Antioxidant Capacity of the Selected Spirulina Extracts During Gastrointestinal Digestion

Depending on the results obtained for bioactive contents of eight different NADES extracts and three different conventional solvent extracts; four of them, including glucose:glycerol (1:2) (NADES 1), betaine:glycerol (1:2) (NADES 5), choline chloride:citric acid (1:2) (NADES 8), and water extracts were selected for further food application and in vitro bioaccessibility and basolateral side recovery measurements. The changes in the TPC and TAC of the selected extracts during in vitro gastrointestinal digestion are represented in Table 2. Gastric digestion significantly decreased the TPCs of all extracts compared to their undigested counterparts (*p* < 0.05). Although intestinal digestion led to slight increases in the TPCs of all extracts after gastric digestion, the changes were not statistically significant (*p* > 0.05). Glucose:glycerol (1:2) (NADES 1) extract exhibited the highest intestinal bioaccessibility of total phenolics (6.05%), which was followed by choline chloride:citric acid (1:2) (NADES 8) (4.89%), water (4.56%), and betaine:glycerol (1:2) (NADES 5) (4.44%) extracts. Similar observations were obtained in another study for specific NADES formulations that yielded the highest initial TPC levels in undigested samples, while representing relatively low bioaccessibility index values post digestion have been attributed to the limited recovery of phenolics under the conditions utilized during digestion and/or possible problems with their quantification (e.g., interactions with other compounds) (Keskin et al. 2025).

**TABLE 2:** Changes in total phenolic and total antioxidant properties of Spirulina extracts during the digestion process.

	TPC (mg GAE/100 mL)			
Extract	Undigested	Gastric digestion	Intestinal digestion	Bioaccessibility index (%)
NADES 1	404.29 ± 48.49^Aa^	13.40 ± 1.50^Ba^	24.24 ± 0.76^Ba^	6.05 ± 0.73^a^
NADES 5	334.76 ± 14.30^Aa^	9.07 ± 2.73^Bb^	14.84 ± 1.43^Bb^	4.44 ± 0.19^b^
NADES 8	303.64 ± 6.97^Ab^	9.85 ± 0.60^Bb^	14.84 ± 1.03^Bb^	4.89 ± 0.11^b^
Water	319.98 ± 9.06^Ab^	11.30 ± 1.43^Bab^	14.59 ± 2.86^Bb^	4.56 ± 0.13^b^
	**CUPRAC (mg TE/100 mL)**			
**Extract**	**Undigested**	**Gastric digestion**	**Intestinal digestion**	**Bioaccessibility index (%)**
NADES 1	327.20 ± 36.54^Aab^	86.58 ± 1.63^Ca^	182.16 ± 1.29^Bb^	56.14 ± 6.31^b^
NADES 5	355.64 ± 31.55^Aa^	86.86 ± 1.02^Ca^	182.71 ± 1.24^Bab^	51.65 ± 4.60^b^
NADES 8	88.03 ± 3.47^Bd^	82.02 ± 0.69^Cb^	181.82 ± 1.37^Ab^	206.76 ± 8.16^a^
Water	288.07 ± 27.92^Abc^	87.08 ± 0.77^Ca^	184.38 ± 1.07^Ba^	64.41 ± 6.27^b^
	**DPPH (mg TE/100 mL)**			
**Extract**	**Undigested**	**Gastric digestion**	**Intestinal digestion**	**Bioaccessibility index (%)**
NADES 1	85.87 ± 10.05^Aa^	22.61 ± 3.16^Bab^	18.13 ± 3.03^Bb^	21.31 ± 2.51^c^
NADES 5	43.88 ± 7.57^Ac^	16.43 ± 2.12^Bc^	14.90 ± 1.20^Bb^	34.65 ± 6.07^ab^
NADES 8	102.56 ± 15.99^Aa^	20.07 ± 1.17^Bbc^	23.96 ± 1.58^Ba^	23.75 ± 3.75^bc^
Water	33.48 ± 3.35^Acd^	25.00 ± 3.85^Aa^	15.07 ± 3.05^Bb^	45.32 ± 4.56^a^

*Note*: Different lowercase letters within the same column indicate statistically significant differences between matrices at the same digestion phase (*p* < 0.05). Different uppercase letters within the same row indicate statistically significant differences among digestion phases (undigested, gastric, and intestinal) within the same sample (*p* < 0.05).

According to the results of CUPRAC and DPPH assays, gastric digestion significantly reduced the antioxidant response of all the tested extracts compared to the undigested samples (*p* < 0.05) (except for the water extract, which had no significant change in the DPPH radical scavenging activity after gastric digestion [*p* > 0.05]). After intestinal digestion, CUPRAC results represented significant increases in comparison to the values obtained after gastric digestion (*p* < 0.05), but they were still significantly lower compared to undigested extracts (*p* < 0.05) (except for NADES 8 extract, which had significantly higher CUPRAC reducing antioxidant capacity after intestinal digestion compared to the undigested sample [*p* < 0.05]). On the other hand, although not statistically significant, intestinal digestion led to further decreases in DPPH results, which may likely be dependent on the degradation or transformation of antioxidants to non‐detectable derivatives under alkaline conditions applied during intestinal digestion (Keskin et al. 2025). CUPRAC assay results represented the highest intestinal bioaccessibility for choline chloride:citric acid (1:2) (NADES 8) extract, with a value exceeding 100% (206.76%), which may be explained by the fact that certain NADES formulations may contribute to the recovery of antioxidant compounds due to enhanced release or transformation to detectable derivatives under digestion conditions (Keskin et al. 2025). On the other hand, the other NADESs and water extracts exhibited decreased intestinal bioaccessibility (ranging between 51.65% and 64.41%), which may suggest possible degradation or interaction effects. The bioaccessible antioxidant response determined via the DPPH method ranged between 21.31% and 34.65% for NADES extracts, which were lower than the value observed for the water extract (45.32%).

### 3.4 Stability of Ready‐to‐Use Spirulina Extracts in Kefir Matrix During Gastrointestinal Digestion

The matrix in which Spirulina extract was incorporated can positively influence the in vitro digestion and the stability of phenolic compounds. Thus, the selected extracts, including NADES 1, NADES 5, NADES 8, and water extracts, were incorporated into the kefir matrix at a concentration of 5% (v/v) and subjected to in vitro gastrointestinal digestion to investigate the changes in phenolic content and antioxidant activity (Table 3).

**TABLE 3:** Changes in TPC and TAC of kefir samples fortified with selected Spirulina extracts during in vitro gastrointestinal digestion.

Kefir + extract	TPC (mg GAE/100 mL)			
	Undigested	Gastric digestion	Intestinal digestion	Bioaccessibility index (%)
Kefir (Control)	2.52 ± 0.41^Cb^	28.61 ± 0.12^Ac^	9.93 ± 0.86^Bc^	—
Kefir + NADES 1	26.30 ± 0.52^Ca^	57.72 ± 4.49^Bb^	93.25 ± 9.49^Aa^	354.65 ± 7.00^a^
Kefir + NADES 5	26.39 ± 0.97^Ba^	60.23 ± 0.57^Aab^	59.61 ± 9.27^Ab^	226.19 ± 8.71^b^
Kefir + NADES 8	26.06 ± 2.56^Ca^	69.18 ± 12.44^Aa^	49.47 ± 2.09^Bb^	191.40 ± 18.76^c^
Kefir + water	25.07 ± 1.40^Ba^	58.09 ± 0.56^Ab^	13.69 ± 1.17^Cc^	54.76 ± 3.09^d^
Kefir + extract	**CUPRAC (mg TE/100 mL)**			
	**Undigested**	**Gastric digestion**	**Intestinal digestion**	**Bioaccessibility index (%)**
Kefir (Control)	11.00 ± 1.41^Cb^	47.55 ± 1.49^Bb^	74.99 ± 4.06^Ad^	—
Kefir + NADES 1	181.60 ± 0.94^Ba^	98.03 ± 7.55^Ca^	260.29 ± 5.61^Aa^	143.33 ± 0.74^a^
Kefir + NADES 5	182.27 ± 2.63^Ba^	98.08 ± 4.68^Ca^	263.84 ± 8.95^Aa^	144.78 ± 2.07^a^
Kefir + NADES 8	180.50 ± 3.47^Ba^	102.19 ± 1.95^Ca^	203.38 ± 4.2^Ab^	112.72 ± 2.18^b^
Kefir + water	183.38 ± 1.82^Aa^	102.19 ± 0.71^Ba^	183.71 ± 2.04^Ac^	100.19 ± 0.99^c^
Kefir + extract	**DPPH (mg TE/100 mL)**			
	**Undigested**	**Gastric digestion**	**Intestinal digestion**	**Bioaccessibility index (%)**
Kefir (Control)	1.01 ± 0.13^Cc^	15.74 ± 1.52^Ba^	19.97 ± 1.00^Ab^	—
Kefir + NADES 1	39.21 ± 20.20^Aa^	11.55 ± 1.84^Bb^	24.36 ± 2.92^ABb^	98.11 ± 31.13^b^
Kefir + NADES 5	21.25 ± 6.11^Aabc^	9.99 ± 1.36^Bbc^	25.35 ± 4.88^Ab^	126.74 ± 31.93^ab^
Kefir + NADES 8	17.79 ± 7.49^Bbc^	7.82 ± 1.57^Cc^	32.45 ± 0.46^Aa^	167.35 ± 51.30^a^
Kefir + water	26.45 ± 7.94^Aab^	8.29 ± 1.87^Bc^	20.21 ± 3.40^Ab^	75.32 ± 17.39^b^

*Note*: Different lowercase letters within the same column indicate statistically significant differences between matrices at the same digestion phase (*p* < 0.05). Different uppercase letters within the same row indicate statistically significant differences among digestion phases (undigested, gastric, and intestinal) within the same sample (*p* < 0.05).

Before digestion, the TPC values determined in the extracts obtained from kefir samples fortified with the selected Spirulina extracts ranged from 25.07 to 26.39 mg GAE/100 mL. These values were lower than those of the corresponding Spirulina extracts before kefir fortification, which may be attributed to the potential interactions of phenolics with kefir components. Phenolic compounds are well reported to form bound phenolics with milk proteins through hydrogen bonding and hydrophobic interactions (Han et al. 2019). Furthermore, the acidic nature of kefir may have enhanced the binding affinity between phenolics and milk proteins (Aiello et al. 2020). Similar to the present study, Mesbah et al. (2022) also observed significantly lower TPC response of Spirulina powder phenolics through its incorporation into yoghurt with a percentage of 1.5%, which resulted in a TPC value of 18.78 mg GAE/100 g fortified yoghurt, compared with 840.60 mg GAE/100 g in the Spirulina powder itself.

Gastric digestion led to significant increases in the TPC of all Spirulina incorporated kefir samples compared to the undigested samples (*p* < 0.05); whereas variable responses were observed among the samples during intestinal digestion. When the samples from intestinal digestion were compared to their counterparts obtained from gastric digestion, kefir sample fortified with glucose:glycerol (1:2) (NADES 1) had significantly higher TPC (*p* < 0.05), no significant change was observed for kefir sample fortified with betaine:glycerol (1:2) (NADES 5) (*p* > 0.05), and significant decreases were observed for both kefir samples fortified with choline chloride–citric acid (1:2) (NADES 8) and with water extracts (*p* < 0.05). All in all, the general trend was a significant increase (*p* < 0.05) in apparent bioaccessible phenolics, as determined by the Folin–Ciocalteu assay, of kefir samples fortified with NADES extracts during gastrointestinal digestion, but kefir added with water extract had significantly lower TPC after digestion (*p* < 0.05). The bioaccessibility index values calculated for TPC of kefir samples fortified with NADES extracts all exceed 100%, with the highest value obtained for NADES 1 extract (354.65%), whereas it was 54.76% for kefir added with water extract. The exceptionally high bioaccessibility index values (exceeding 100%) observed through the Folin–Ciocalteu assay may partly be attributed to the additional release of protein‐complexed phenolics (those that provided higher TPC values in original NADES extracts [before having complexes with kefir proteins] than in kefir samples fortified with NADES extracts) in the kefir matrix during digestion. It has previously been reported that strong phenolic–food matrix (protein, fiber, etc.) interactions may result in incomplete extraction of many phenolics, and this may be overcome under digestion conditions with the influence of several factors, such as pH and enzymatic hydrolysis, leading to more efficient recovery of these compounds during digestion (Yan et al. 2018). These substantial increases in bioaccessibility index values were also attributed to the increases in detectable phenolics, likely due to release or transformation of these compounds under digestion conditions (Keskin et al. 2025). But it should also not be overlooked that this assay is not specific only to phenolic compounds and may also respond to other reducing substances (i.e., citric acid, simple sugars, amino acids) originating from NADES or released during digestion that may interfere with the results and lead to overestimation (Capanoglu et al. 2022).

The possible release of peptides and amino acids from the kefir matrix during digestion may affect the results of spectrophotometric measurements. The Folin–Ciocalteu reagent may react with amino acids such as tyrosine, tryptophan, and cysteine (Everette et al. 2010), and kefir matrix contains antioxidative amino acids, including methionine, glutamine, and lysine (Ozcan et al. 2019). In addition, the interaction of polyphenols with proteins depends on both amino acid composition and phenolic structure, and the formation of polyphenol–protein complexes may alter the availability and apparent response of bioactive compounds (Perna et al. 2019). Therefore, the increased TPC response observed after digestion may partly reflect the release of matrix‐derived amino acids and the dissociation of phenolic–protein complexes. This interpretation is also consistent with the study of La Torre et al. (2024), who reported that antioxidant capacity in kefir samples was not necessarily correlated with TPC, suggesting that non‐phenolic contributors may also affect the measured antioxidant response.

Furthermore, the TPC results observed during gastrointestinal digestion also indicated that the solvent system may substantially affect the apparent Folin‐reactive responses measured in the intestinal phase. The post‐digestion increase in TPC levels has previously been explained by the fact that NADES may contribute to the facilitated release of bound phenolics during digestion through carrying the bound phenolic‐matrix complexes and protecting them from degradation under digestion conditions (Bekar 2025).

Gastric digestion led to significantly lower values in both CUPRAC and DPPH assays compared to undigested samples (*p* < 0.05). On the other hand, intestinal digestion significantly increased the antioxidant responses measured by both assays in comparison to the samples from gastric digestion (*p* < 0.05). According to CUPRAC results, the apparent bioaccessible antioxidant response ranged in between 112.72% (for kefir fortified with NADES 8) and 144.78% (for kefir fortified with NADES 5) for kefir samples fortified with NADES extracts, and this index was 100.19% for kefir added with water extract. The bioaccessibility index values determined using DPPH results that exceed 100% were observed for kefir samples fortified with NADES 8 (167.35%) and NADES 5 (126.74%). Similar to the TPC results, these values should not be interpreted as direct evidence of true antioxidant absorption. Differences between DPPH and CUPRAC responses may arise from their distinct analytical principles, since DPPH reflects radical scavenging activity, whereas CUPRAC is based on copper (II)‐reducing capacity. In protein‐rich kefir samples, amino acids, peptides, and other reducing compounds released during digestion may contribute particularly to electron transfer‐based assays such as CUPRAC (Ozkan et al. 2026).

### 3.5 Changes in TPC and TAC of Ready‐to‐Use Spirulina Extracts During Caco‐2 Cell Transport

Before conducting transport experiments, the maximum nontoxic sample concentrations were determined, and TEER measurements of the cells treated with these predetermined nontoxic sample concentrations were performed (Table S3) in order to ensure that the cell integrity was protected during the analysis. Mitochondrial metabolic activity (MTT) assay was performed with diluted digests at ratios of 1/2, 1/5, 1/10, and 1/20 to determine the nontoxic sample concentration. A minimum of 80% cell viability was observed at a dilution ratio of 1/2 for water extract, NADES 1, and NADES 5 extracts; at a dilution ratio of 1/5 for NADES 8 extract; and at a dilution ratio of 1/10 for all kefir samples fortified with the selected extracts.

**FIGURE 2:**
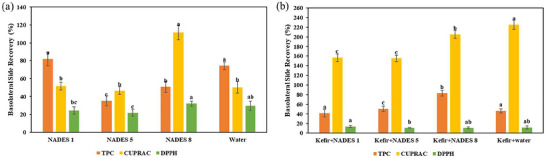
Apparent basolateral recovery of TPC, CUPRAC, and DPPH responses in selected Spirulina extracts (a) and Spirulina‐fortified kefir samples (b) after Caco‐2 cell transport. Different lowercase letters indicate statistically significant differences among extracts within the same analysis (*p* < 0.05).

The highest apparent basolateral recovery of TPC among the NADES extracts was observed for NADES 1 (81.88%), followed by the water extract (74.33%). Also, the highest apparent basolateral recovery measured by the CUPRAC assay was obtained for NADES 8 (111.86%) (Figure 2). The corresponding CUPRAC‐based recoveries of the other extracts ranged from 46.66% to 51.55%, while the DPPH‐based response ranged in between 22.03% and 32.31%.

In Spirulina‐fortified kefir samples, the highest apparent basolateral recovery of TPC was detected in kefir supplemented with NADES 8 extract (82.97%), whereas the highest CUPRAC response was observed in kefir supplemented with water extract (225.05%), followed by kefir supplemented with NADES 8 (205.20%), NADES 1 (156.49%), and NADES 5 (155.01%) extracts. The DPPH radical scavenging response of the basolateral fractions of fortified kefir samples ranged from 10.89% to 13.81%. These findings indicate that matrix incorporation significantly affected the apparent transepithelial transport of antioxidants.

During the intestinal phase, the protein–phenolic complexes in kefir are hydrolyzed due to factors such as pH and digestive enzymes, thereby releasing bound phenolics and free amino acids from the matrix that give rise to a higher response compared to Spirulina extracts. Higher apparent basolateral recoveries measured in Spirulina incorporated kefir samples, compared to individual Spirulina NADES extracts, through CUPRAC assay (and partly through TPC assay), may partly be explained by the release of reactive amino acid units from the kefir matrix during Caco‐2 cell utilization. This consideration was also supported by Guidea et al. (2020), who indicated that electron transfer‐based methods such as CUPRAC assay may be influenced by compounds other than phenolics, including amino acids. Similarly, Everette et al. (2010) reported that the Folin–Ciocalteu reagent used for TPC determination is not specific to phenolics and may also react with amino acids like tyrosine, tryptophan, and cysteine. Thus, the elevated apparent TPC and CUPRAC recoveries observed in fortified kefir samples may partly reflect the contribution of matrix‐derived amino acids and other reducing substances in addition to phenolic‐related compounds.

The combined use of simulated gastrointestinal digestion and the Caco‐2 monolayer model is widely accepted as a cost‐effective in vitro approach for mimicking physiological conditions, but it cannot fully replicate in vivo conditions, because it does not account for the complexity of whole‐body metabolism, chronic dietary exposure, or gut microbiota‐mediated transformations (Prado Massarioli et al. 2023; Redan et al. 2017). Moreover, spectrophotometric methods may not fully reflect the actual bioactivity of antioxidants under biological conditions; however, these methods are considered suitable for minimizing the complexity of biological systems and providing comparative antioxidant values for preliminary screening (Martinelli et al. 2021).

## 4 Conclusion

This study demonstrated the capacity of different NADES compositions for the recovery of Spirulina bioactives, both from the original biomass and after incorporation into a food matrix, during extraction and in vitro gastrointestinal digestion. Among the tested NADES systems, glucose:glycerol yielded the highest chlorophyll (27.67 mg/100 mL), phycocyanin (519.98 mg/100 mL), TPC (404.29 mg GAE/100 mL), and DPPH (85.87 mg TE/100 mL) values. While the recovery of antioxidants from Spirulina biomass during gastrointestinal digestion was generally low, their recovery in kefir was higher, which was partly attributed to the protective effect of the acidic nature of the kefir matrix. These findings may support the idea that food matrices not only stabilize phenolic compounds during digestion but also enhance their release and absorption. The results further suggest that a dairy‐based matrix may help protect certain phenolic compounds from degradation during gastric and intestinal digestion, thereby enhancing their release and subsequent absorption. Although the findings support the potential use of kefir as a carrier matrix for ready‐to‐use NADES‐based Spirulina extracts, further studies should investigate different food matrices and processing conditions and evaluate sensory acceptance, formulation feasibility, and practical applicability under realistic consumer and industrial conditions. In addition, the use of compound‐specific analytical approaches and in vivo models is needed to confirm whether the apparent Caco‐2 transport responses observed in this study correspond to the actual intestinal absorption of Spirulina‐derived phenolics.

## Author Contributions

**Gamze Toydemir**: writing – original draft, writing – review and editing, formal analysis, data curation. **Ilayda Sanli**: writing – original draft, data curation, formal analysis, writing – review and editing, methodology. **Gulay Ozkan**: conceptualization, writing – review and editing, methodology, data curation, writing – original draft. **Esra Capanoglu**: supervision, conceptualization, writing – review and editing.

## Conflicts of Interest

The authors declare no conflicts of interest.

## Supporting information




**Supplementary Material**: jfds71289‐sup‐0001‐SuppMat.docx
