# Supplementary material for: Ultrasound‐Assisted NADES‐Based Extraction of Spirulina Bioactives: Bioaccessibility and Intestinal Permeability Assessment Using an In Vitro Digestion/Caco‐2 Cell Model and Food Application
Source: J Food Sci. 2026 Jul 17;91(7):e71289. doi: 10.1111/1750-3841.71289 (PMC13377542; doi:10.1111/1750-3841.71289)
Supplement: Supplementary file 1 — Supplementary Material: jfds71289‐sup‐0001‐SuppMat.docx [file JFDS-91-0-s001.docx]

**Table S1.** The compositions and densities of the NADES solutions

| **NADES** | **NADES composition** | | | | **Molar ratio** | **Density**  **(kg/m^3^)** |
| --- | --- | --- | --- | --- | --- | --- |
|  | **Component 1** | **Component 2** | **Component 3** | **Component 4** |  |  |
| 1 | Glucose | Glycerol | Water | ─ | 1:2:2 | 1129 ± 1^b^ |
| 2 | Choline chloride | Lactic acid | Water | ─ | 1:2:2 | 1067 ± 0^d^ |
| 3 | Betaine | Lactic acid | Water | ─ | 1:2:2.5 | 1065 ± 2^d^ |
| 4 | Glycerol | Lactic acid | Water | ─ | 1:1:2.5 | 1047 ± 1^e^ |
| 5 | Betaine | Glycerol | Water | ─ | 1:2:2 | 1077 ± 1^c^ |
| 6 | Betaine | Glycerol | Glucose | Water | 1:2:4:0.5 | 1190 ± 0^a^ |
| 7 | Choline chloride | Acetic acid | Water | ─ | 1:1:2 | 1041 ± 2^f^ |
| 8 | Choline chloride | Citric acid | Water | ─ | 1:2:1.5 | 1187 ± 1^a^ |

*Different lowercase letters within the same column indicate statistically significant differences among sample densities (p < 0.05).

**Table S2.** Absorbance values of solvent blanks (NADES and conventional solvents) used for TPC, DPPH, CUPRAC, and pigment assays

| **Solvent** | **TPC (A765)** | **DPPH (A517)** | **CUPRAC (A450)** | **Pigment (A664)** | **Pigment (A440)** | **Pigment (A652)** | **Pigment (A647)** | **Pigment (A620)** |
| --- | --- | --- | --- | --- | --- | --- | --- | --- |
| NADES 1 | 0.075±0.002 | 0.358±0.006 | 0.128±0.008 | 0.049±0.001 | 0.055±0.001 | 0.049±0.001 | 0.049±0.001 | 0.049±0.001 |
| NADES 2 | 0.077±0.001 | 0.239±0.012 | 0.103±0.001 | 0.050±0.001 | 0.056±0.000 | 0.050±0.001 | 0.050±0.001 | 0.050±0.001 |
| NADES 3 | 0.062±0.000 | 0.337±0.004 | 0.103±0.001 | 0.050±0.001 | 0.057±0.001 | 0.050±0.001 | 0.051±0.001 | 0.051±0.001 |
| NADES 4 | 0.071±0.002 | 0.339±0.011 | 0.109±0.001 | 0.048±0.003 | 0.054±0.001 | 0.048±0.002 | 0.049±0.002 | 0.049±0.002 |
| NADES 5 | 0.061±0.001 | 0.357±0.007 | 0.131±0.003 | 0.052±0.002 | 0.058±0.003 | 0.052±0.002 | 0.052±0.002 | 0.053±0.003 |
| NADES 6 | 0.076±0.001 | 0.325±0.031 | 0.189±0.011 | 0.052±0.002 | 0.063±0.001 | 0.052±0.002 | 0.052±0.002 | 0053±0.001 |
| NADES 7 | 0.063±0.002 | 0.329±0.014 | 0.110±0.003 | 0.061±0.001 | 0.078±0.001 | 0.061±0.001 | 0.062±0.001 | 0.063±0.002 |
| NADES 8 | 0.064±0.000 | 0.194±0.004 | 0.103±0.003 | 0.051±0.001 | 0.059±0.001 | 0.051±0.001 | 0.051±0.001 | 0.052±0.001 |
| Methanol (80%) | 0.058±0.002 | 0.353±0.035 | 0.109±0.001 | 0.051±0.002 | 0.057±0.001 | 0.051±0.002 | 0.052±0.002 | 0.052±0.003 |
| Ethanol (80%) | 0.059±0.004 | 0.370±0.029 | 0.109±0.002 | 0.045±0.001 | 0.051±0.002 | 0.045±0.001 | 0.046±0.001 | 0.046±0.001 |
| Water | 0.051±0.003 | 0.357±0.005 | 0.108±0.001 | 0.046±0.002 | 0.050±0.001 | 0.046±0.002 | 0.047±0.002 | 0.047±0.002 |

Absorbance values of solvent blanks were measured under identical assay conditions and subtracted from the corresponding sample absorbance before calculation of these assays.

**Table S3.** TEER values (Ω·cm²) before and after transport experiments

| **Sample** | **Before transport** | **After transport** |
| --- | --- | --- |
| Control | 265±18 | 245±15 |
| NADES 1 | 268±12 | 257±14 |
| NADES 5 | 241±20 | 231±17 |
| NADES 8 | 275±8 | 245±11 |
| Water | 239±13 | 223±8 |
| Kefir + NADES 1 | 283±10 | 255±13 |
| Kefir + NADES 5 | 230±16 | 210±19 |
| Kefir + NADES 8 | 279±9 | 275±12 |
| Kefir + Water | 247±19 | 218±5 |

* TEER values were corrected by subtracting the blank (medium) value (11 ± 2 Ω·cm²).

**Table S4.** Exact p-values obtained from ANOVA

| **Pigment composition (One-way ANOVA)** | | | | | |
| --- | --- | --- | --- | --- | --- |
| **Parameter** | | | **p-value** | | |
| Chlorophyll | | | p < 0.001 | | |
| Carotenoids | | | p < 0.001 | | |
| Phycocyanin | | | p < 0.001 | | |
| **Digestion Experiments (Two-way ANOVA – Matrix × Digestion)** | | | | | |
| **Parameter** | **Matrix effect** | | **Digestion effect** | **Matrix × Digestion** | |
| TPC | p < 0.001 | | p < 0.001 | p < 0.001 | |
| CUPRAC | p < 0.001 | | p < 0.001 | p < 0.001 | |
| DPPH | p < 0.001 | | p < 0.001 | p < 0.001 | |
| **Digestion Phase Comparisons within Each Sample (One-way ANOVA)** | | | | | |
| **Sample** | **TPC (p-value)** | | **CUPRAC (p-value)** | **DPPH (p-value)** | |
| NADES 1 | p < 0.001 | | p < 0.001 | p < 0.001 | |
| NADES 5 | p < 0.001 | | p < 0.001 | p < 0.001 | |
| NADES 8 | p < 0.001 | | p < 0.001 | p < 0.001 | |
| Water | p < 0.001 | | p < 0.001 | p = 0.145 | |
| Kefir (control) | p < 0.001 | | p < 0.001 | p < 0.001 | |
| NADES 1 + Kefir | p < 0.001 | | p < 0.001 | p = 0.005 | |
| NADES 5 + Kefir | p < 0.001 | | p < 0.001 | p < 0.001 | |
| NADES 8 + Kefir | p < 0.001 | | p < 0.001 | p < 0.001 | |
| Water + Kefir | p < 0.001 | | p < 0.001 | p < 0.001 | |
| **Bioaccessibility and Basolateral Recovery (One-way ANOVA)** | | | | | |
| **Bioaccessibility index** | | | **Transport efficiency** | | |
| **Parameter** | | **p-value** | **Parameter** | | **p-value** |
| TPC (extract) | | p = 0.003 | TPC (extract) | | p < 0.001 |
| CUPRAC (extract) | | p < 0.001 | CUPRAC (extract) | | p < 0.001 |
| DPPH (extract) | | p = 0.001 | DPPH (extract) | | p < 0.001 |
| TPC (kefir + extract) | | p < 0.001 | TPC (extract + kefir) | | p < 0.001 |
| CUPRAC (kefir + extract) | | p < 0.001 | CUPRAC (extract + kefir) | | p < 0.001 |
| DPPH (kefir + extract) | | p = 0.006 | DPPH (extract + kefir) | | p = 0.038 |

**Table S5.** Chromatographic characterization of major phenolic compounds in Spirulina extracts obtained with NADES and conventional solvents

|  | Gallic acid | Syringic acid | 2,3,4-Trihydroxybenzoic acid | (-)-Epicatechin | Rutin | Quercetin | Apigenin | Chlorogenic acid | Ferulic acid |
| --- | --- | --- | --- | --- | --- | --- | --- | --- | --- |
| NADES 1 | 542.64±28.91^a^ | 0.92±0.27^cde^ | 0.30±0.01^bc^ | 2.80±0.19^cd^ | 1.21±0.11^bc^ | 0.23±0.03^a^ | 0.42±0.07^ab^ | Nd | 1.26±0.07^ab^ |
| NADES 2 | 55.62±16.53^b^ | Nd | 0.30±0.13^bc^ | 10.60±0.53^a^ | Nd | Nd | Nd | Nd | 0.92±0.13^b^ |
| NADES 3 | 37.24±1.44^b^ | Nd | Nd | 10.20±2.18^a^ | Nd | Nd | 0.33±0.11^b^ | Nd | 0.90±0.16^b^ |
| NADES 4 | 39.88±2.71^b^ | 1.28±0.02^cde^ | Nd | 3.06±0.43^cd^ | Nd | Nd | Nd | Nd | 0.70±0.21^b^ |
| NADES 5 | 567.21±26.24^a^ | 2.53±0.36^b^ | 0.96±0.13^a^ | 7.37±0.62^ab^ | 1.10±0.03^c^ | 0.23±0.04^a^ | 0.37±0.05^b^ | Nd | 1.28±0.24^ab^ |
| NADES 6 | 414.40±42.69^a^ | 1.62±0.17^bc^ | Nd | 3.42±0.02^cd^ | 1.37±0.32^bc^ | 0.18±0.02^a^ | 0.26±0.01^b^ | Nd | 0.86±0.04^b^ |
| NADES 7 | 112.58±18.52^b^ | 0.65±0.11^cde^ | Nd | 2.06±0.32^cd^ | Nd | Nd | 0.71±0.14^ab^ | Nd | 1.09±0.27^ab^ |
| NADES 8 | 505.95±68.47^a^ | 0.21±0.06^e^ | 0.11±0.01^c^ | 3.17±0.90^cd^ | Nd | Nd | Nd | 1.04±0.30^a^ | Nd |
| Methanol | 74.52±18.34^b^ | 1.16±0.15^cde^ | Nd | 1.48±0.25^d^ | 1.22±0.03^bc^ | 0.34±0.08^a^ | 0.91±0.27^a^ | 0.38±0.05^b^ | 1.96±0.49^a^ |
| Ethanol | 23.57±4.64^b^ | 0.42±0.01^de^ | 0.74±0.12^ab^ | 3.48±0.81^cd^ | 2.46±0.28^a^ | Nd | 0.51±0.04^ab^ | Nd | 1.95±0.43^a^ |
| Water | 462.28±101.02^a^ | 3.77±0.59^a^ | 0.99±0.27^a^ | 5.30±0.54^bc^ | 1.98±0.22^ab^ | 0.27±0.01^a^ | 0.60±0.18^ab^ | Nd | 1.11±0.02^ab^ |

*Different lowercase letters within the same column indicate statistically significant differences (p < 0.05).


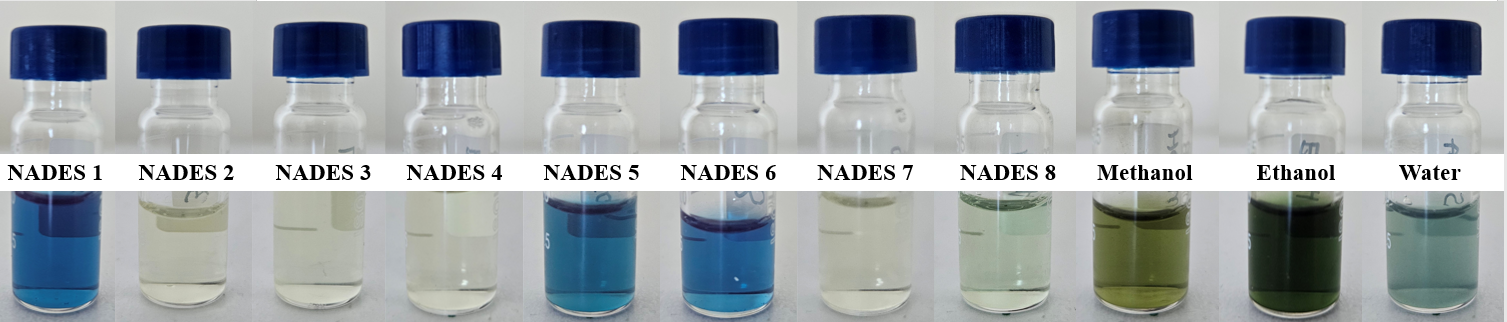
**Figure S1**. Appearance of Spirulina extracts obtained using conventional solvents and NADES systems after ultrasound-assisted extraction.
